# A 10-nm Spectral Resolution Hyperspectral LiDAR System Based on an Acousto-Optic Tunable Filter

**DOI:** 10.3390/s19071620

**Published:** 2019-04-04

**Authors:** Yuwei Chen, Wei Li, Juha Hyyppä, Ning Wang, Changhui Jiang, Fanrong Meng, Lingli Tang, Eetu Puttonen, Chuanrong Li

**Affiliations:** 1 Center of Excellence of Laser Scanning Research, Finnish Geospatial Research Institute, Masala FI-02430, Finland; yuwei.chen@nls.fi (Y.C.); juha.hyyppa@nls.fi (J.H.); chiang_changhui@outlook.com (C.J.); eetu.puttonen@nls.fi (E.P.); 2 Key Laboratory of Quantitative Remote Sensing Information Technology, Chinese Academy of Sciences (CAS), Beijing 100094, China; wangning@aoe.ac.cn (N.W.); mengfanrong@aoe.ac.cn (F.M.); lltang@aoe.ac.cn (L.T.); crli@aoe.ac.cn (C.L.); 3 School of Automation, Nanjing University of Science and Technology, Nanjing 230013, China

**Keywords:** hyperspectral LiDAR, supercontinuum laser (SCL), acousto-optic tunable filter (AOTF), system calibration, vegetation analysis

## Abstract

Hyperspectral LiDAR (HSL) technology can obtain spectral and ranging information from targets by processing the recorded waveforms and measuring the time of flight (ToF). With the development of the supercontinuum laser (SCL), it is technically easier to develop an active hyperspectral LiDAR system that can simultaneously collect both spatial information and extensive spectral information from targets. Compared with traditional LiDAR technology, which can only obtain range and intensity information at the selected spectral wavelengths, HSL detection technology has demonstrated its potential and adaptability for various quantitative applications from its spectrally resolved waveforms. However, with most previous HSLs, the collected spectral information is discrete, and such information might be insufficient and restrict the further applicability of the HSLs. In this paper, a tunable HSL technology using an acousto-optic tunable filter (AOTF) as a spectroscopic device was proposed, designed, and tested to address this issue. Both the general range precision and the accuracy of the spectral measurement were evaluated. By tuning the spectroscopic device in the time dimension, the proposed AOTF-HSL could achieve backscattered echo with continuous coverage of the full spectrum of 500–1000 nm, which had the unique characteristics of a continuous spectrum in the visible and near infrared (VNIR) regions with 10 nm spectral resolution. Yellow and green leaves from four plants (aloe, dracaena, balata, and radermachera) were measured using the AOTF-HSL to assess its feasibility in agriculture application. The spectral profiles measured by a standard spectrometer (SVC^©^ HR-1024) were used as a reference for evaluating the measurements of the AOTF-HSL. The difference between the spectral measurements collected from active and passive instruments was minor. The comparison results show that the AOTF-based consecutive and high spectral resolution HSL was effective for this application.

## 1. Introduction

The concept of hyperspectral LiDAR (HSL) emerged at the beginning of this century [1], combining the active hyperspectral imaging technique and LiDAR distance measuring technique into a single instrumental framework with fine spectral resolution. Such integration also efficiently resolves the registration problems between the hyperspectral images collected by hyperspectral imagers and the point clouds collected by LiDAR systems. The quantitative and qualitative analysis capabilities of HSL are undoubtedly enhanced by combining the geometric information with spectral information from its spectrally resolved waveforms. Such point cloud data with additional multiple spectral information create innovative opportunity for automatic point cloud processing, target classification, and environmental understanding [2,3,4,5].

The combination of hyperspectral imaging and LiDAR into a single framework has been studied in the remote sensing community for decades. Point clouds collected by active LiDAR fused with spectral images collected by passive imaging spectrometry have been extensively investigated for various research purposes, such as forest inventory, land classification, and mineral identification. These researches supported the conclusion that fusion of the spatial information from active LiDAR and the spectral images from the passive imager presented better performance than the methods based on any single sensor [6,7,8]. For example, the Academy of Opto-Electronics (AOE), Chinese Academy of Sciences (CAS), proposed a scheme that combined the monochrome laser source based pushbroom-style LiDAR and the spectrometric imaging sensor by sharing an optical system to quickly generate 3D spectral images [9,10]. However, the data processing for combination of the two datasets is complex due to the registration problem. Moreover, this system was not immune to the changes in illumination conditions and had a restricted ability to preclude the shadow region because the spectrometric images were collected in a passive manner, which could affect the final detection results [11].

To achieve hyperspectral records of the return laser signal, multiple lasers of different wavelengths or a supercontinuum laser (SCL) are the current major choices. With multiple lasers operating on different wavelengths, researchers can easily build hyperspectral LiDARs, especially for dual-wavelength LiDAR systems for agriculture and forestry related research [12,13,14,15]. Furthermore, the research team from University of Edinburgh proposed a 4-wavelength multispectral canopy LiDAR with four lasers operating on 531 nm, 550 nm, 660 nm, and 780 nm for virtual forest monitoring. The designed 4-wavelength multispectral LiDAR was used to detect the normalized vegetation index (NVDI) and photochemical vegetation index (PRI) for vegetation detection [16]. Wuhan University also developed a multi-wavelength canopy LiDAR system that operates at four wavelengths over a range most sensitive to the retrieval of vegetation characteristics from four semiconductor laser diodes subsequently synthesized into a single beam. This LiDAR is mainly used in vegetation remote sensing and object classification [3,4,17,18]. The advantage of a multiple lasers based solution is that the system can be quickly built with commercial off-the-shelf (COTs) products for proof-of-concept studies. Generally, this type of HLS system consists of a combination of several standalone LiDARs operating on different wavelengths and sharing the same optical system. However, the disadvantages of a multiple lasers based solution are also obvious: (1) the spectral band is redistricted by the availability of the monochrome laser source, resulting in limited versatility; (2) the laser emitting system becomes bulky as the number of channels increases; (3) the synthesis of multiple laser sources into a single beam requires considerable efforts in system design, especially if additional spectral band configuration is anticipated.

Use of SCL is an alternative option for HSL. Normally, a commercial SCL continuously covers the visual to short-wavelength infrared (SWIR) spectrum, which is ideal for applications involving continuous spectral coverage. However, with SCL, a spectroscopic device is needed to spectrally separate the broadband laser signal. Such device can consist of optical filters [19,20,21], a spectrograph [2], a liquid crystal tunable filter (LCTF) [22], and an acousto-optic tunable filter (AOFT) [23]. The Finnish Geospatial Research Institute (FGI) has performed research on HSL technology based on SCL sources for forestry applications since 2007 [1]. In 2010, the FGI demonstrated an SCL-based two-channel hyperspectral LiDAR using optical filters [19], and in 2012, the FGI created the first SCL-based full waveform HSL system using a spectrograph to simultaneously generate eight spectral signals in the range of 450–1000 nm [2]. The AOE designed an 8-channel HSL covering the visible to SWIR spectrum range (450–1600 nm) using optical filters as the spectroscopic device and extended it to a 17-channel version for ore classification, and the result was promising [20,21]. The AOE also designed a LCTF-based HSL system (referred to as LCTF-HSL) to detect the red edge for vegetation-related application, and the comparison results showed that the red-edge positions extracted from it were similar to spectrometer measurement [23]. The LCTF-HSL offered continuous spectrum coverage, but had limited spectrum range of the LCTF component, together with a low power density of the transmitting broadband laser at 450–550 nm. The effective range was close, and only data from 18 spectral channels were available for the appropriate signal-to-noise ratio from 550 nm with 10 nm spectral resolution. Another potential disadvantage was the tuning speed of the LCTFs, which is normally several tens of milliseconds and is mainly determined by the switching speed of the liquid crystal elements. The low tuning speed could restrict mobile and airborne applications in the future. Furthermore, multiple LCTFs in separate optics paths were required in the optical designs for the hyperspectral LiDAR systems.

In general, most of the developed HSLs have the characteristics of limited and discrete spectral channels, which strongly restrict understanding of the novelty of the technology and hinders further applicability. To tackle this problem, this paper proposes, designs, and tests a tunable HSL technology based on an AOTF device known as AOTF-HSL. Due to the applied AOTF component, the complexity, dimensions, and hardware investment of HSL are considerably decreased, and a continuous spectrum with a finer spectral resolution of 10 nm can be achieved. AOTFs offer a quicker tuning speed (microseconds) and broader wavelength ranges compared with the LCTF for HLS application [24]. Additionally, multiple simultaneous frequencies can be applied to the AOTF device without excessive intermodulation. Such characteristics allow controlled wavelength mixing, which is preferable for spectral channel selection for a dedicated HSL application.

The major contributions of this research are listed as follows:

(1) The AOTF-HSL solution is proposed, designed, and tested in laboratory conditions. The designed AOTF-HSL operates on a spectrum range from 500 nm to 1000 nm with a 10 nm spectral resolution. The instrument represents an advancement related to previous similar instruments in allowing genuinely hyperspectral (continuous) data to be generated rather than discrete multispectral data. The new system also represents a significant advance in terms of the number of channels available. The laser emission unit consists of an SCL and an AOTF device, ensuring that different wavelengths of the laser beam can be emitted at each time slot. This design allows continuous wavelength selection of the laser pulse in the time dimension and also the filtered pulse at the exit of the transmitted component for better eye safety. Additionally, such a time-multiplexing solution is different with spectrograph based HSL.
(2) Distance measurement capabilities are marginally addressed with a basic demonstration and simple precision assessment. The range precision or range stability over 51 spectral channels of the AOTF-HSL is preliminarily evaluated with a simple test case, four standard reflection boards (100%, 70%, 40%, and 20%) placed at a measured distance of 37.5 meters, and the random range errors can be observed.
(3) In order to precisely calibrate the HSL intensity, the corresponding relationship between the target attribute and the output waveform of HSL is determined based on analysis of the radiation transmission mechanism of HSL, and the calibration model and method are constructed for more accurate quantitative applications of HSL. LiDAR calibration can be achieved by passing a sample of the transmitted signal through the receiver and monitoring the signal from the standard targets both from a spectrometer and the HSL. The calibration tests are conducted using different standard diffuse reflectors (99%, 70%, 40%, 20%, and 5%).
(4) The AOTF-HSL application to agriculture is verified by various vegetation experiments. The spectral profiles of green and yellow leaves from four species are analyzed. The calibrated AOTF-HSL experimental results are also compared with the corresponding measurements from a standard spectrometer (SVC^©^ HR-1024) (hereinafter referred to as the SVC spectrometer). The average spectral difference (absolute value) of the six leaf samples are minor, i.e., 1.37% (green leaf of dracaena), 8.79% (yellow leaf of dracaena), 0.84% (green leaf of aloe), 4.6% (yellow leaf of aloe), 1.36% (green leaf of balata), and 0.77% (green leaf of radermachera), indicating that the AOTF-based high spectral resolution HSL is effective for this application. The results reveal that the potential of this active remote sensing is applicable for vegetation research.

The remainder of the paper is organized as follows. Section 2 introduces the details of the AOTF-HSL system design, and Section 3 presents the testing and calibration of the laboratory experiments. The results are discussed in Section 4, and conclusions are presented in Section 5.

## 2. Methods 

The AOTF is a tunable bandpass narrow-band filter with a bandwidth of several to tens of nanometers that uses the acousto-optic effect to diffract and shift the frequency of light using usually a radio frequency (RF) signal. The selected wavelength is determined by Equation (1)

(1) $$\lambda =\frac{\Delta nV}{f}{\left[\sin^{2}2\theta_{i}+\sin^{4}\theta_{i}\right]}^{1/2}$$

where the selected wavelength (*λ*) is a function of the difference of the refractive indices due to birefringence ($\Delta n=n_{i}-n_{d}$), the frequency of the applied RF signal (*f*), the speed of acoustic waves in the crystal material (*V*), and the incident angle ($\theta_{i}$) between the source laser beam and the crystal material to be modulated in the radio frequency.

In this research, an AOTF device was used to rapidly and dynamically select a specific wavelength from the broadband white laser source. As the applied RF frequency on the AOTF device is varied, the filtered wavelength changes to generate consecutive and high-spectral-resolution laser pulses in time dimension, and the tuning of the wavelength normally requires tens of microseconds or less, which is ideal for terrestrial and mobile HSL development for better future operational efficiency. Non-simultaneous measurements on different bands seem to be the inherent disadvantage of an AOTF based HSL solution. However, it is not problematic given the high tuning speed of the filter, and it spends half a millisecond to acquire the full spectrum with current full spectrum configuration when the synchronization between the AOTF device and broadband laser source can be setup.

**Figure 1:**
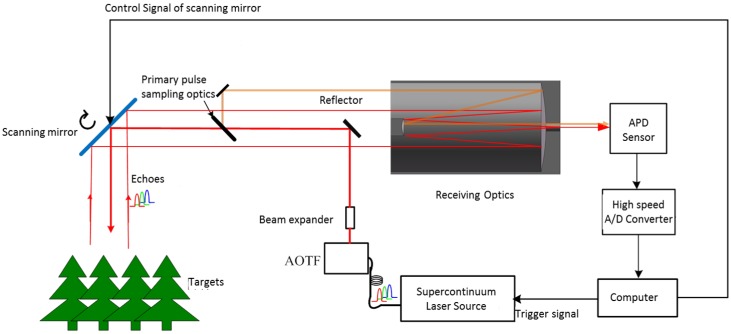
Schematic setup of acousto-optic tunable filter based hyperspectral lidar (AOTF-HSL).

The AOTF-HSL was designed based on the schematic illustration in Figure 1. A computer triggers a laser source to transmit a “white” laser pulse. The laser source is an SCL source (model YSL^®^ SC-OEM, YLS photonics Inc., Wuhan, China) with a wavelength ranging from 450 to 2,400 nm, and the maximum power of a single pulse exceeds 1.9 µJ. The pulse rate of the SCL is 0.01–1 MHz with a 2–3 ns full width at half maximum (FWHM) over the wavelength range [25]. The “white” laser pulse is emitted from a microstructure optical fiber (MOF) and is filtered by the AOTF device (model YSL^®^ AOTF-Pro, YLS photonics Inc., Wuhan, China), which is a wavelength selection module based on the acousto-optic crystal. The technical specifications of the chosen AOTF module can be found in Table 1. A beam expander (×5) is used to collimate the filtered beam with a high divergence angle before transmission to the object. Primary pulse sampling optical components are installed in the transmitted optics path to direct a trivial portion of the transmitted pulse to the receiving optics. The collected signal is applied to trigger a high-speed A/D converter to archive the waveforms of both the transmitting pulse and the receiving echoes on the selected spectral band, and thus the time-of-flight and spectral intensity can be measured by applying post-processing algorithms on the collected waveform. The collimated narrow-band laser beam is reflected towards the target via the reflector placed at the optical axis of the receiving telescope. A scanning mirror controlled by the computer points the transmitted narrow-band laser beam to the targets. A Cassegrain telescope (with 700 mm focal length and 100 mm aperture diameter) collects the scattered laser pulse from the targets. The focal point of the telescope is imaged onto a high-voltage-biased low-noise-level avalanche photodiode (APD) model (MenloSystems APD210, Menlo Systems GmbH, Munich, Germany), and the model consists of a silicon APD and an integrated amplifier with a bandwidth of 1 GHz, which converts the laser echo into an electronic signal and amplifies it. The output signals from the APD model are sampled and recorded using a high-speed oscilloscope with a 10 GHz sampling rate. The major difference between the AOTF-HSL and other HSLs is that a single pixel APD is used rather than an APD array to collect the return in the time dimension when a specific wavelength is filtered from the SCL by the AOTF device. The AOTF-HSL tunes its outputs with 10 nm spectral resolution, resulting in a set of 51 channels with spectrally resolved waveform echoes covering 500–1000 nm for the sampling points on the target. When all spectrally resolved waveform echoes on the current sampling point are collected, the computer turns the scanning mirror to the next sampling point and repeats the full measurement procedure again.

## 3. Tests and Calibration

Based on the schematic setup introduced in Section 2, the AOTF-HSL was designed and tested in a laboratory environment (corridor of the 9th floor of the AOE main building). Even though the APD model was not fully covered by shields, the potential impact of the varying ambient illumination conditions was still minor due to the fact that the oscilloscope collected the echo signal in AC coupling mode. Two tests were conducted to generally evaluate the range precision and the precision of the spectral profile. The targets used in the range test included four standard grey boards with 100%, 70%, 40%, and 20% reflectivity, respectively. Yellow and green leaves from four plants, namely, aloe (*Aloe arborescens Mill.*), dracaena (*Dracaena angustifolia*), balata (*Ficus elastica Roxb. ex Hornem.*) and radermachera (*Radermachera hainanensis Merr.*), were used in spectral measurement evaluation by comparing the corresponding measurements from the SVC spectrometer.

### 3.1. Range Precision Test

Due to the complexity of the AOTF-HSL optical design, the authentic range cannot be easily measured with the current setup. Thus, the range precision or stability was evaluated instead of range accuracy by measuring the identical range of four standard grey boards (20%, 40%, 70%, and 100%) with perpendicular laser beams. The range was 37.50 m measured by a commercial Leica laser telemeter (Leica D8) by measuring the range between the receiving optics (Cassegrain telescope) and the targets, which was then used as the reference range rather than the genuine value to evaluate the range performance.

The grey boards were placed at a distance of more than 37.50 m from the AOTF-HSL to test the uniformity of the range measurements over the spectral channels. For each grey board, the center point of the grey board was selected for range measurement because of perpendicular laser beams. The range precision was evaluated based on range measurements covering the full spectrum (51 spectral channels). For each spectral channel, 16 measurements (waveforms) were collected at the same conditions and the range result of each spectral channel is the average results of its 16 measurements.

The time-of-flight measurements of different spectral channels were calculated based on the collected waveforms recorded at 10 G samplings per second with a simple maximum algorithm.

### 3.2. Spectral Profile Test

Aloe (*Aloe arborescens Mill.*), dracaena (*Dracaena angustifolia*), balata (*Ficus elastica Roxb. ex Hornem.*) and radermachera (*Radermachera hainanensis Merr.*) plants were selected for the spectral profile test due to their diversity on the properties of the leaves. The continuous spectral profiles with 10 nm resolution were collected from six different leaves (four green samples and two yellow samples) of four species plants in a range from 500 nm to 1000 nm assisted by a white reflectance standard with 99% reflectivity. The collected spectral profiles were compared with the reference data collected by the SVC spectrometer. The distance between the plants and the AOTF-HSL was approximately 6 meters to collect better signal-to-noise ratio waveforms, especially in the 500–650 nm range.

### 3.3. Calibration of the Spectral Profile

The precision of the intensity calibration of the HSL system is a prerequisite for many quantitative applications, and it has recently become an important object of study [26,27]. In addition to the range, the AOTF-HSL has the ability to record the target reflected powers in a series of continuous spectral channels. Therefore, calibration must be conducted before we can accurately reveal the physical essence behind the spectral observations. The LiDAR equation, which summarizes all of the parameters relevant to describe the process of signal receiving by a LiDAR sensor, can be written as shown [26]:

(2) $$P_{r}=\frac{P_{t}D_{r}^{2}}{4\pi R^{4}\beta_{t}^{2}}\sigma$$

where $P_{r}$ is the power entering the receiver, $P_{t}$ is the transmitted power, $D_{r}$ is the aperture diameter of the receiver optics, R is the range, $\beta_{t}$ is the transmitter beam width, and σ is the backscatter cross-section, which can be expressed as follows:

(3) $$\sigma =\frac{4\pi }{\Omega }\rho A_{S}$$

where Ω is a cone of a solid angle into which the incoming radiation is scattered uniformly, ρ is the surface reflectance, and $A_{S}$ is the receiving area of the scatter.

**Figure 2:**
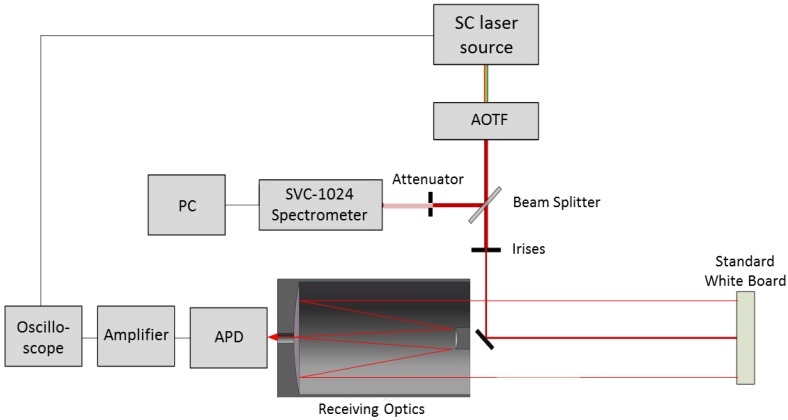
Calibration of AOTF-HSL.

LiDAR calibration can be achieved by passing a sample of the transmitted signal through the receiver and monitoring the signal from the standard targets [28]. A similar method can also be used in the airborne laser scanning system with portable brightness targets due to the Lambertian reflectance characteristics and known reflectance values [29,30]. In this paper, the intensity of the AOTF-HSL was calibrated in the laboratory with a Spectralon^®^ (Labsphere Inc., North Sutton, NH, USA) white reflectance standard as the reference. The entire calibration system is illustrated in Figure 2.

In calibration, the output energy of the laser beam of the selected spectral channel from the AOTF is monitored by the SVC spectrometer via a beam splitter (T:R = 92:8) and an attenuator (8%). A diaphragm is used to filter out the higher-order diffracted laser beam for better accuracy. A white reflectance standard with 99% reflectivity is used as the target for calibration. From the LiDAR equation, we find that once the calibration system is set up, the backscatter cross-section of the white reflectance standard, the aperture diameter of the receiver optics, and the range and the transmitter beam width are all unchanged. From the SVC spectrometer measurement, the transmitted power can be calculated. Assuming that the response of the APD ($f_{APD}(\lambda )$) is a linear model, the response curve of the APD (V(λ)) on a selected wavelength (*λ*) can be modelled by the following Equation (4).

(4) $$f_{APD}\left(\lambda \right)=aV\left(\lambda \right)+b$$

where the coefficient of the linear model and the variable b denote the offset of the model. By modifying the transmission power of the SCL source to change the transmitted power, the response curves of both the ADP and spectrometer can be achieved. Thus, both the coefficients of the linear model and the offsets of the linear model for all spectral channels can be determined.

## 4. Results and Discussion

### 4.1. Range Precision Evaluation

**Figure 3:**
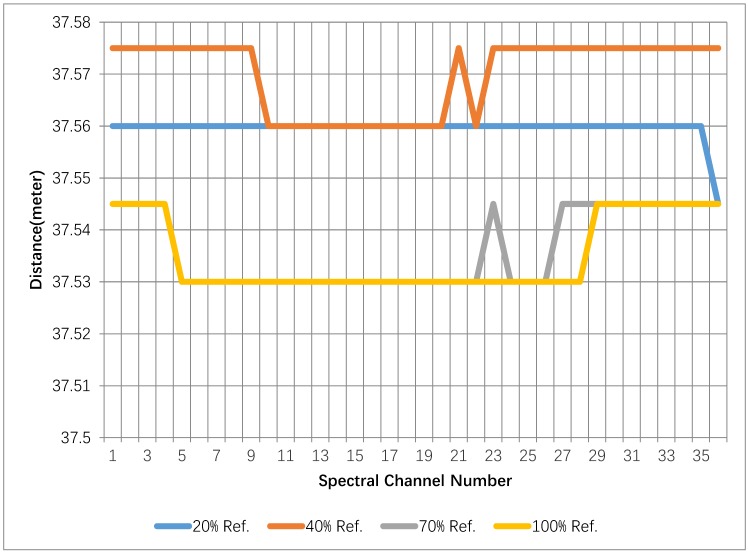
Range measurements of the AOTF-HSL from 650 nm to 1000 nm on 4 standard targets with different reflectivity.

The range is calculated based on the simple maximum algorithm by subtracting the peak position of the echo pulse with the corresponding peak positioning of the primary pulse from the original waveform. It can be observed from Figure 3 that the range measurements of the standard grey boards differ from 37.53 m to 37.575 m and that the variances of the range measurements over 36 channels are 2.5 mm (20%), 7.2 mm (40%), 7.5 mm (70%), and 7.1 mm (100%), respectively. Promising uniformity of the range measurements over spectral channels can be observed for selected grey boards.

One issue that must be addressed is that due to the current AOTF-HSL system configuration, the authentic value of the range to the standard reflectivity board is not available. It can be concluded from the conducted range evaluation that with 10 G samplings per second the derived range resolution from the ToF measurements for different bands is 1.5 centimeters, which might be insufficient for some applications, and thus more advanced post-processing algorithms should be investigated to improve the range resolution. The random range error (maximum of 45 mm) from different reflectivity boards should also be mitigated by more sophisticated waveform processing if better range precision is needed, e.g., some forms of averaging algorithm to increase SNR, and Gaussian fitting. However, the tendency observed is that the range measurement increases with the decrease in the reflection of the target. In Figure 3, the measurements of the spectral channels from 500–650 nm are missing due to the low sensitivity of the APD sensor and the low transmitted power intensity of the SCL below 650 nm [2].

### 4.2. Spectral Profile Calibration

**Figure 4:**
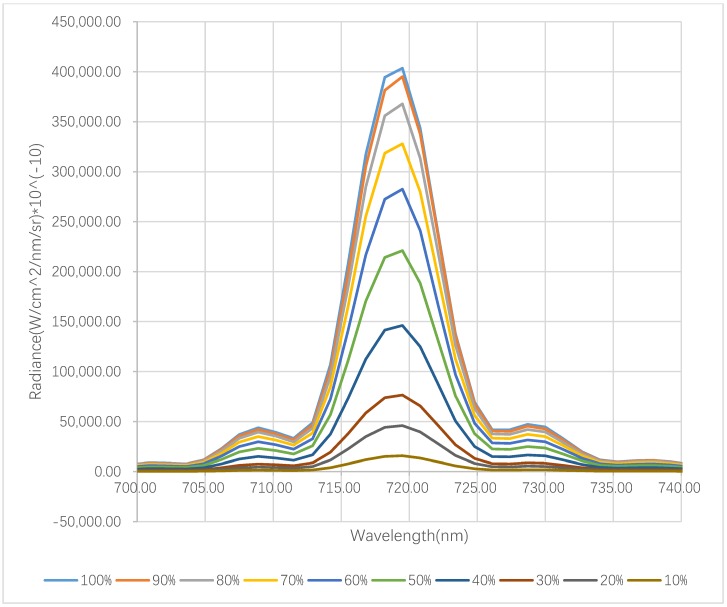
Output of the SVC^®^ spectrometer by modifying the transmission power of the supercontinuum laser (SCL) source from 10–100% with a 10% step length at 720 nm on a white reflectance standard with 99% reflectivity.

Figure 4 shows the SVC spectrometer measurements related to the different transmitted power levels of the SC laser at 720 nm. The transmission power of the SCL source is modified from 10% to 100% with a 10% step length. It can be seen from Figure 4 that the main lobe of the recorded measurements follows an approximately Gaussian distribution and the coefficients of determination (R^2^) of the 10 transmitted power levels are better than 0.973. The center wavelength of each record is close to 720 nm. Additionally, the associated side lobes can be discerned, and the amplitude of the side lobe is approximately 1/10 of that of 720 nm.

**Figure 5:**
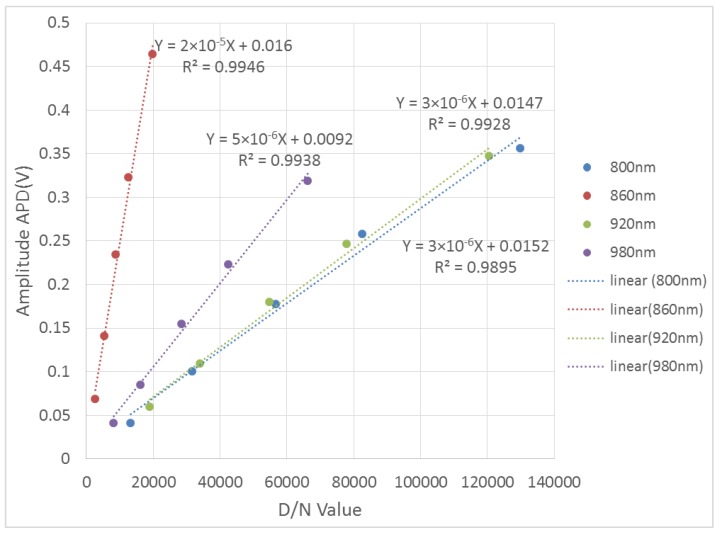
Relationship between AOTF-HSL measurement and spectrometer measurement in selected NIR bands.

The relationship between the APD measurements (maximum amplitude of the return echo) and the spectrometer measurements (D/N value) at each spectral channel can be built up. Figure 5 presents the selected results for 800 nm, 860 nm, 920 nm, and 980 nm, respectively, for the with five reflectance standards with 99%, 70%, 40%, 20%, and 5% reflectivity. Good linearity between the AOTF-HSL measurements and the spectrometer measurements can be observed, and the coefficients of determination (R^2^) of the four selected spectral channels are better than 0.993. The fitting residuals are 0.025661, 0.022756, 0.019263, and 0.017471 respectively due to the sensitivity variations between the charge coupled device (CCD) employed by the spectrometer and the APD sensor, which proves that the proposed calibration method is applicable. Such a calibration method is used in the intensity calibration of the AOTF-HSL in the following section.

### 4.3. Spectral Profiles

**Figure 6:**
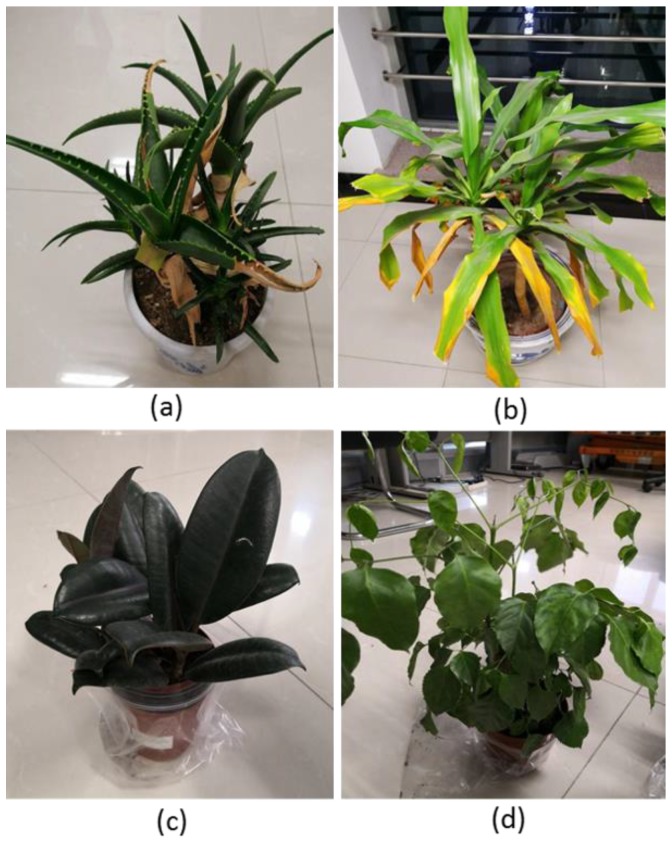
Four plants under test: (**a**) aloe with yellow and green leaves, (**b**) dracaena with yellow and green leaves, (**c**) balata and (**d**) radermachera.

For the spectral profile test cases, all plant targets shown in Figure 6 were measured at a 6-meter distance to ensure that all spectral channels can collect the reliable S/N ratio original waveforms because the sensitivity of the Si-APD and the laser intensity of the selected SCL below 650 nm are low. The reference spectral profiles were collected by the SVC spectrometer using sunlight as the light source and were acquired at a 25-cm distance.

**Figure 7:**
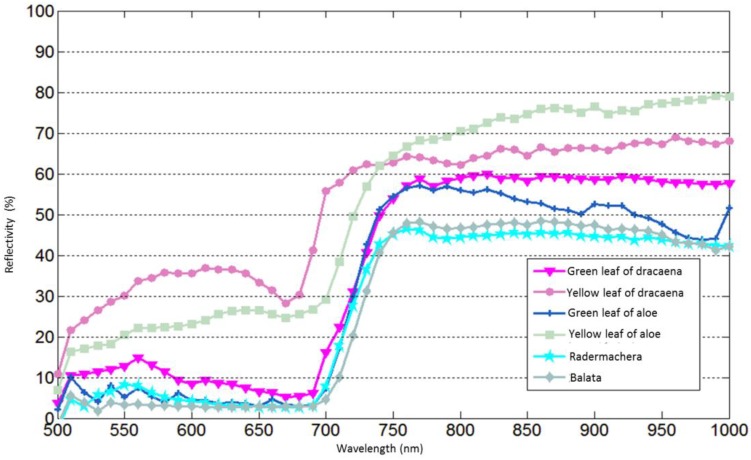
Hyperspectral Lidar derived spectral profiles of 6 leaves from 4 plant species (dracaena, aloe, balata, and radermachera).

**Figure 8:**
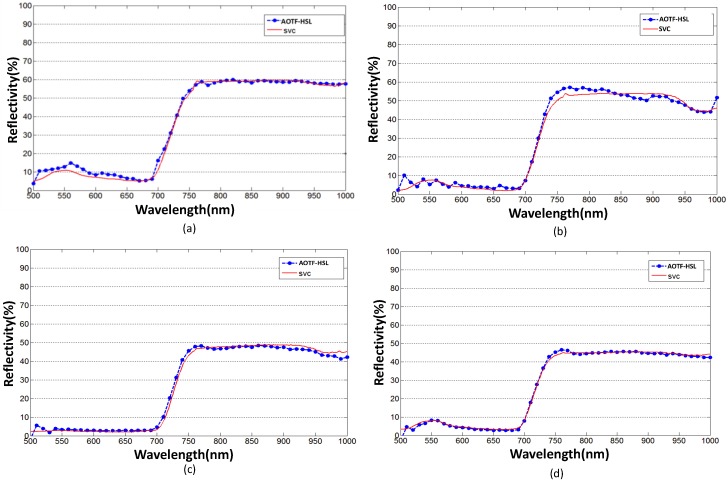
HSL derived spectral profile of green leaves from (**a**) dracaena (**b**) aloe, (**c**) balata (**d**) radermachera compared with spectrometer measurements.

**Figure 9:**
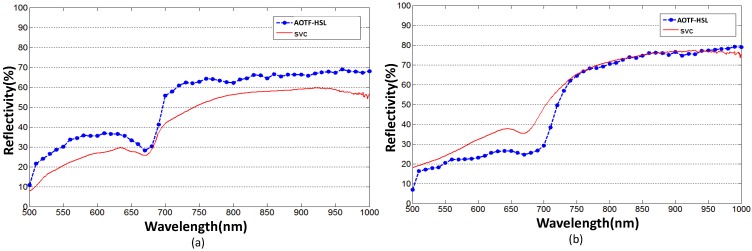
HSL derived spectral profile of yellow leaves from (**a**) dracaena, (**b**) aloe compared with spectrometer measurements.

The reflectivity of each spectral channel is calibrated based on the method presented in Section 4. Figure 7 shows the spectral profiles for six leaf samples from four species of plants. The “red edge” of four green leaf cases can be easily discerned. For the yellow leaf cases, higher reflectivity can be observed compared with the corresponding green leaf cases due to the loss of chlorophyll. Four spectral profiles collected by the AOTF-HSL from the green leaves of each plant are presented in Figure 8 against the reference curves collected by the spectrometer, and we see that with the proposed calibration method, the results from the AOTF-HSL are more comparable to the spectrometer result. The active HSL and passive spectrometer spectral profiles clearly coincide, especially for the “red edge” referring to the rapid change region of vegetation reflectance or an inflection point of the reflectance slope in the red band spectrum [31,32]. The average spectral differences (absolute value) of four green leaves for active and passive measurements are 1.37% (dracaena), 0.84% (aloe), 1.36% (balata), and 0.77% (radermachera), respectively. Figure 9 presents the spectral profiles collected from the yellow leaves of dracaena and aloe against the corresponding spectrometer measurements. The average spectral differences are larger than those of the green leaf cases, specifically, 8.79% for dracaena and 4.6% for aloe.

It can be observed from Figure 8 and Figure 9, that the backscattered reflectivity produced by the HSL does not strictly follow the reference values. Three reasons may cause the discrepancies as a synthesized result of multiple factors. First, the illuminated surface area of HSL (the diameter of the footprint is 12 mm) is smaller than the sampled area of the spectrometer (resulting in a 5.5 cm radius footprint with a 25° field of view for the selected module), and such a mismatch might introduce major differences, especially if the non-uniformity of the yellow leaf is greater than that of the green leaf. Second, the uniformity of the green leaf is better than that of the yellow leaf, and thus the location of the sampling area is critical, especially for the yellow leaf cases, and a minor difference exists between the two measurements. Third, the transmitted pulse energy of the SCL source might vary slightly, and according to the specifications of the laser source, the power stability of the SCL source is better than 1% [25]. Some modifications in the optics system should be done to mitigate the discrepancies in the reflectance estimation, such as adding a beam expander in the exit of the broadband laser source to match the footprint size between the two systems.

**Figure 10:**
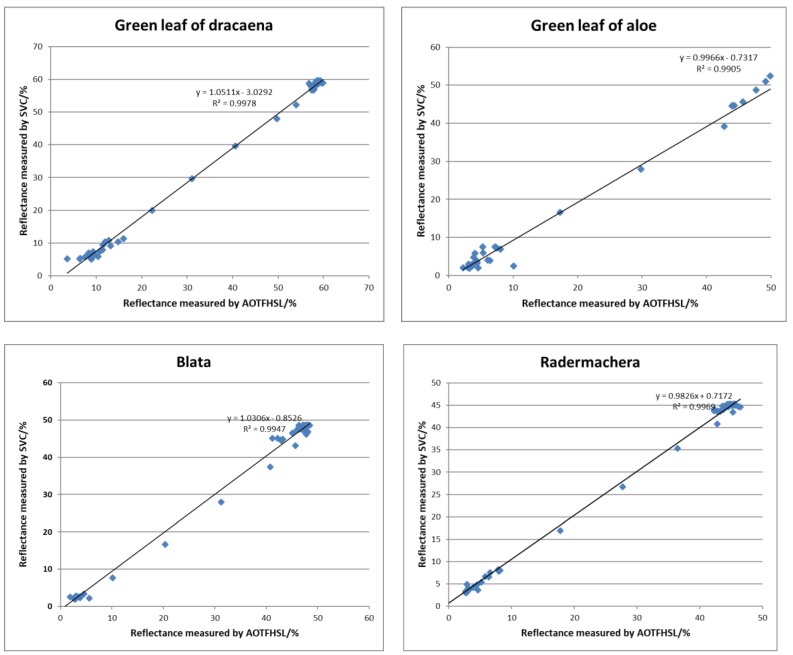
Scatter diagrams of the reflectance of green leaf cases measured by AOTF-HSL and SVC spectrometer.

**Figure 11:**
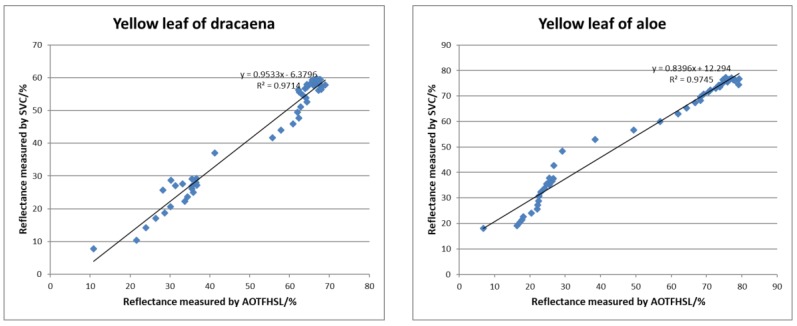
Scatter diagrams of the reflectance of yellow leaf cases measured by AOTF-HSL and SVC spectrometer.

We computed the distributions of the reflectance from the green leaves of the four plants from the hyperspectral LiDAR, compared them with the reflectance determined by the spectrometer data, and present the scatterplots of the reflectance from AOTF-HSL versus that from the SVC spectrometer in Figure 10 with a linear fit. Higher coefficients of determination (R^2^ > 0.99) in all four cases indicate that the extracted reflectance from HSL correlates highly with the referenced results. For the four green leaf cases the average value is close to 0.9949, which demonstrates the excellent fitness of the measurements from the two devices. Similar scatter diagrams of the yellow leaf cases for dracaena and aloe are illustrated in Figure 11, and compared with the green leaf cases the coefficients of determination are poorer (R^2^ > 0.97) but still promising. It can be preliminarily concluded that the reflectance results of the HSL system using the proposed calibration method are reliable.

## 5. Conclusions

This work presented the general abilities in range and continuous spectral profile collection for a designed AOTF-based 51-channel HSL with 10-nm spectral resolution. The range precision could reach tens of millimeters, and the effective range of the prototyped HSL could reach several tens of meters, at least for spectral channels higher than 650 nm with the current system configuration, which is highly promising for terrestrial and mobile applications. Using the calibration method of passing a sample of the transmitted signal through the receiving optics and monitoring the spectrometer signal from the standard targets, we could more accurately calibrate the spectral profile collected by the AOTF-HSL. The AOTF-HSL application to agriculture was verified by vegetation experiments. The spectral profiles of green and yellow leaves from four species were analyzed. The calibrated AOTF-HSL experimental results were also compared with the corresponding measurements from a standard spectrometer. The average spectral difference (absolute value) of the leaf samples is minor. The comparison of the spectral profiles collected with the HSL and the spectrometer showed reliable fitness, which proved that the reflectance results of the HSL system were trustworthy.

Our future work will improve the applicability of the system by assessing the proper range of accuracy throughout the whole spectrum and by designing a terrestrial/mobile version AOTF-HSL for additional field tests rather than tests in a controlled laboratory environment, e.g., integration of a georeferenced system and altitude measurement devices. Additionally, other types of applications, such as environmental understanding based on geometrical characteristics and spectral features for robotics, will also be investigated with the current configuration to verify its generic applicability.

## Figures and Tables

**Table 1:** Specifications of the designed acousto-optic tunable filter based hyperspectral Lidar (AOTF-HSL).

Parameter	
Spectral range	430–1450 nm
Spectral resolution	2–10 nm
Output efficiency	>40%
Polarization	Line polarization
Beam divergence	0.4 mill radian
Beam diameter (at exit)	10 mm
